# Implementing high-value, cost-conscious care: experiences of Irish doctors and the role of education in facilitating this approach

**DOI:** 10.1186/s12909-024-05666-x

**Published:** 2024-06-21

**Authors:** Evan Carroll, Crisann Tan, Samantha Hayes, Serge Mordang, Gabriella Rizzo, Victor Zaia, Erik Montagna, Karen D. Könings, Anél Wiese, Colm O’Tuathaigh

**Affiliations:** 1 School of Medicine, University College Cork, Cork, Ireland; 2 Educational Development and Research, School of Health Professions Education, Faculty of Health, Medicine and Life Sciences, Maastricht University, Maastricht, The Netherlands; 3 Department of Medicine, Cork University Hospital, Cork, Ireland; 4 Faculdade de Medicina do ABC, Centro Universitário FMABC, Santo André, SP Brazil; 5 Medical Education Unit, School of Medicine, University College Cork, Cork, Ireland; 6 Maastricht University Office, Maastricht University , Maastricht, Netherlands

**Keywords:** High-value cost-conscious care, Undergraduate medical education, Junior doctors, Medical specialists

## Abstract

**Background:**

Adopting high-value, cost-conscious care (HVCCC) principles into medical education is growing in importance due to soaring global healthcare costs and the recognition that efficient care can enhance patient outcomes and control costs. Understanding the current opportunities and challenges doctors face concerning HVCCC in healthcare systems is crucial to tailor education to doctors’ needs. Hence, this study aimed to explore medical students, junior doctors, and senior doctors’ experiences with HVCCC, and to seek senior doctors’ viewpoints on how education can foster HVCCC in clinical environments.

**Methods:**

Using a mixed-methods design, our study involved a cross-sectional survey using the Maastricht HVCCC-Attitude Questionnaire (MHAQ), with a subset of consultants engaging in semi-structured interviews. Descriptive analysis provided insights into both categorical and non-categorical variables, with differences examined across roles (students, interns, junior doctors, senior doctors) via Kruskal-Wallis tests, supplemented by two-group analyses using Mann-Whitney U testing. We correlated experience with MHAQ scores using Spearman’s rho, tested MHAQ’s internal consistency with Cronbach’s alpha, and employed thematic analysis for the qualitative data.

**Results:**

We received 416 responses to the survey, and 12 senior doctors participated in the semi-structured interviews. Overall, all groups demonstrated moderately positive attitudes towards HVCCC, with more experienced doctors exhibiting more favourable views, especially about integrating costs into daily practice. In the interviews, participants agreed on the importance of instilling HVCCC values during undergraduate teaching and supplementing it with a formal curriculum in postgraduate training. This, coupled with practical knowledge gained on-the-job, was seen as a beneficial strategy for training doctors.

**Conclusions:**

This sample of medical students and hospital-based doctors display generally positive attitudes towards HVCCC, high-value care provision, and the integration of healthcare costs, suggesting receptiveness to future HVCCC training among students and doctors. Experience is a key factor in HVCCC, so early exposure to these concepts can potentially enhance practice within existing healthcare budgets.

**Supplementary Information:**

The online version contains supplementary material available at 10.1186/s12909-024-05666-x.

## Introduction

Incorporating high-value, cost-conscious care (HVCCC) principles into medical education is becoming increasingly important [1]. This shift in focus is driven by the escalating costs of healthcare globally and the growing recognition that efficient, effective care can both improve patient outcomes and help control these costs [2]. High-value, cost-conscious care is a philosophy that strives for optimal patient care while taking into account associated costs [3]. It aims to curb unnecessary healthcare spending, such as redundant treatments or overtesting, with a view to either lowering overall healthcare costs without compromising care quality or increasing quality of care based on the same costs [3]. Given the global rise in healthcare expenses, HVCCC is becoming a central topic in healthcare and medical education reform discussions. Healthcare expenditure has witnessed a substantial surge worldwide, with spending in the European Union reaching €1.073 billion, equivalent to 8% of the GDP, in 2020 [4, 5]. Concurrently, the United States recorded healthcare costs of $8.5 trillion in 2019, constituting 9.8% of its GDP. Meanwhile, Ireland’s healthcare budget rose to €26.5 billion in 2020, reflecting an 11% growth from 2019, and a considerable 29% escalation since 2013 [6]. For this reason, emphasis has been placed on devising a more efficient strategy for healthcare system spending, with the goal of curbing costs while maintaining the quality of patient care, aspects that are fundamental principles of HVCCC [7].

Doctors hold a pivotal position in implementing HVCCC and they are uniquely situated to contribute to improvements in healthcare cost management [8]. They are continuously engaged in decisions about tests, treatments, and procedures for their patients, and they play a critical role in educating patients about their treatment options, including related costs. The core principles of HVCCC should be guiding these decisions, which entails weighing not only the prospective benefits and risks of an intervention but its cost-effectiveness as well [9]. Furthermore, doctors bear the professional responsibility of staying informed about studies concerning the cost-effectiveness of various interventions, as well as evidence about overused or low-value care, and have a duty to oversee healthcare resources conscientiously, considering this information.

Despite doctors’ potential to champion HVCCC, several challenges persist. These include a lack of training and awareness about HVCCC among doctors, pressure to meet patient expectations that may conflict with HVCCC, a non-supportive culture within healthcare organisations, limited access to timely and accurate cost information, absence of standardised guidelines or insufficient evidence on high-value care, and the fear of malpractice lawsuits leading to defensive medicine [10, 11].

Medical education plays a vital role in addressing the challenges associated with implementing HVCCC [7]. Educational initiatives like the Choose Wisely campaign, which started in North America in 2012 and has since been embraced by numerous countries globally, have had some success in fostering nationwide discussions between healthcare providers and patients about avoiding unnecessary tests, procedures, and treatments [12]. Additionally, international medical education bodies have established competencies that define the roles and responsibilities of doctors in relation to HVCCC [13, 14].

Until recently, it appears that education focused on HVCCC has predominantly targeted postgraduate training and continuing professional development [15]. Nonetheless, there is a growing acknowledgement that introducing HVCCC principles early on allows medical students to cultivate an understanding of the balance between cost and care quality, thereby better equipping them for real-world medical practice [16]. However, current undergraduate medical programmes do not adequately focus on HVCCC, potentially leading to graduates with limited competence regarding healthcare economics [17]. This signals a demand for curricular development that incorporates learning about healthcare expenses and value-oriented care. To effectively revise curricula, it is essential to first understand the present opportunities and obstacles doctors encounter regarding HVCCC implementation in healthcare systems. This will aid in identifying learning needs and equipping future doctors to apply HVCCC principles effectively. For these reasons, this study sought to investigate the attitudes and experiences of medical students, recently qualified (i.e. interns) and junior doctors, as well as senior doctors toward HVCCC. It also aimed to understand the perspectives of senior doctors on the role of education, both undergraduate and postgraduate, in enhancing HVCCC implementation in clinical settings.

## Methods

### Study design

This study used a mixed-methods approach rooted in a pragmatic epistemological stance. This approach emphasises practical outcomes and the value of using multiple methods to comprehensively address research aims. Our decision to combine both quantitative and qualitative methods was driven by the desire to leverage the distinct benefits each method offers, leading to a more comprehensive understanding of our participants’ experiences with HVCCC. Initially, a quantitative survey was conducted enabling us to gather extensive data, capturing a wide scope of experiences and attitudes toward HVCCC across a large participant pool and to identify overall patterns. Subsequently, qualitative semi-structured interviews afforded us an in-depth exploration of individual narratives to delve deeper into these patterns and obtain a more nuanced understanding of the personal perspectives and experiences surrounding HVCCC.

To provide context for this study, the Irish healthcare system is a mix of public and private where the system is mostly tax-financed, but where supplementary private health insurance (held by approximately 40% of the population) and out-of-pocket expenses are also used to pay for a significant amount of healthcare costs [18].

### Participants and setting

Participants belonged to one of the following four groups: students at an Irish medical school; interns completing their internship across the six geographically-defined Irish intern training networks; junior doctors (i.e. non-consultant hospital doctors) from a variety of medical and surgical specialties across ten teaching hospitals affiliated with University College Cork (UCC) School of Medicine (https://www.hse.ie/eng/about/who/acute-hospitals-division/hospital-groups/south-southwest/); senior doctors from all specialties across the the same hospitals.

This study was approved by the Social Research Ethics Committee of University College Cork (Reference Number: 29/07/2020).

### Data collection

Survey: All participants were asked to complete the Maastricht HVCCC-Attitude Questionnaire (MHAQ) [19], alongside a range of socio-demographic and career-oriented questions. The MHAQ, a validated 25-item tool used in this study, evaluates attitudes towards HVCCC in various healthcare settings using a four-point Likert scale (from ‘strongly disagree’ to ‘strongly agree’). Subscales in the MHAQ, stemming from the original validation study, included “Provision of High Value Care” (8 items), “Integration of Healthcare Costs” (10 items), and “Drawbacks of HVCCC” (7 items). These subscales assess attitudes about doctors’ roles in providing high-value care, the integration of healthcare costs in daily practice, and perceived challenges of implementing HVCCC respectively. Cronbach’s alpha values between 0.61 and 0.82 on all subscales have previously been reported for doctors and patients [19].

Eligible participants were invited to anonymously complete the questionnaire through a web-based Microsoft Forms link. Specifically, medical students were contacted by the Medical School office with the invitation to complete the survey. Medical interns were invited via email to participate by the South Intern Training Network administrator. A random sample of 150 junior and 50 senior doctors were contacted via email by the Medical School office to participate in this study. Data collection took place from September 2020 to May 2021.

Semi-structured interviews: Our qualitative study design was constructed in alignment with the Consolidated Criteria for Reporting Qualitative Research (COREQ) to ensure the research design’s robustness [20]. A semi-structured interview template was developed to assess opinions related to HVCCC, including obstacles, enablers, and education, based on findings from the current and previously reported MHAQ questionnaire data and a comprehensive literature review [19, 21–23] (Supplementary Data 1). An initial pilot interview was conducted with a general practitioner affiliated with the medical school to assess the overall clarity and wording of the questions included in the interview guide, timing of the interview, and usefulness of follow-up and probing questions. The template was not changed based on this pilot exercise.

We invited ten senior doctors to participate in the interview via email through purposive sampling while employing convenience and snowball sampling techniques to recruit an additional two participants. The concept of data saturation, which refers to the point where interviews no longer yield new information, guided the decision-making process regarding when to cease data collection [24]. This approach ensured that the collected data was rich and comprehensive enough to accurately represent the phenomenon being studied and draw meaningful conclusions. Our participant group consisted of twelve consultant doctors operating in three distinct hospitals in southwest Ireland, across eight medical and surgical specialties to ensure a representative view. Consultants are fully trained doctors in a specific medical field, equivalent to attending doctors in North America. This experience is beyond the postgraduate training, which typically lasts four to six years in Ireland, depending on the specialty. We chose to focus our interviews on consultant-level doctors, as their seniority and experience within our national healthcare system position them to provide the most insightful perspectives on HVCCC implementation issues. Their critical role in both undergraduate and postgraduate medical education, coupled with their responsibilities in directly mentoring students and junior doctors, makes them well-suited to provide insights on leveraging education to enhance HVCCC effectively.

Amid the COVID-19 pandemic and the resulting restrictions, the interviews were carried out through various virtual platforms such as phone calls, Microsoft Teams, and Zoom. Two researchers (EC, CO’T) were responsible for conducting these interviews, which occurred between December 2020 and June 2022.

### Data analysis

Summary descriptive analysis was completed for categorical and non-categorical variables. Group differences based on position/role (medical student, intern, junior doctor, senior doctor) was examined by Kruskal-Wallis test, with two-group post-hoc analyses, as well as other two-group independent groups (e.g. gender) comparisons, conducted using Mann-Whitney U testing. The association between experience (i.e. years since graduation) and MHAQ scores was tested using Spearman’s rho test of correlation. We also tested internal consistency reliability using Cronbach’s alpha for the MHAQ instrument. For this study, a conventional two-tailed P value of 0.05 was employed. All analyses were conducted using IBM SPSS version 26.0.

The audio recordings were transcribed verbatim. For analysis, these transcriptions were imported into NVivo 12 [25]. We employed Braun and Clarke’s thematic analysis approach to identify recurring themes within the interview transcripts [26]. Initially, the 12 interviews were collectively analysed to improve familiarity with the data. We used open coding to create initial codes that were continually adjusted and refined throughout the process. Similar codes were grouped together to form distinct themes. These themes were then carefully reviewed and analysed to ensure accurate representation of the data and to prevent over interpretation. The formal definition of each theme and the collaboration of results occurred after validation. This process of theme identification was carried out by two investigators (EC, CO’T).

## Results

### MHAQ results

416 responses were received, distributed across the following professional categories: medical student (63%, *N* = 262; a response rate of 25% based on a total eligible population of 1045 students), intern (19%, *N* = 80; a response rate of 10% based on a total eligible population of 804 medical interns), junior doctor (14%, *N* = 57; a response rate of 38%), and senior doctor (4%, *N* = 16; a response rate of 32%), with females making up 57.6% of respondents. A summary of the demographic and educational/professional characteristics information is available in Table 1. Cronbach’s alpha values for MHAQ (total, subscale) scores are presented in Table 2; what is considered a low alpha value (< 0.6) was only observed for the “Drawbacks of HVCCC” MHAQ sub-scale in the medical student sample. In the full sample, individual item ratings (4-point scale, Strongly Disagree to Strongly Agree) were highest for “Provision of High Value Care” (mean = 3.14, SD = 0.37), followed by “Integration of Healthcare Costs” (mean = 2.69, SD = 0.56), and “Drawbacks of HVCCC” (mean = 2.47, SD = 0.48).

**Table 1:** Summary of demographic and educational characteristics, as well as mean (SD) MHAQ item scores, for medical students, interns, junior doctors, and senior doctors

	Medical student N = 262	Intern N = 80	Junior Doctor N = 57	Senior Doctor N = 16	Total N = 416
**Age** (mean no. of years, SD)	22.6 (3.4)	26.0 (2.7)	31.5 (6.0)	49.7 (6.1)	25.6 (6.9)
**Gender** (%)					
Male	39.2	41.3	51.0	68.8	42.4
Female	60.7	58.7	49.0	31.2	57.6
Other	0	0	0	0	0
**Nationality** (%)					
Irish	40.5	71.3	78.9	56.3	52.3
Overseas	32.8	28.7	21.1	43.7	30.8
Not stated	26.7	0	0	0	16.9
**Years since graduation** (mean no. of years, SD)	-	1.4 (0.9)	6.6 (5.5)	21.8 (8.25)	-
**Mode of entry to medical school** (%)					-
Undergraduate entry Graduate entry	61.3 38.7	73.1 26.9	67.3 22.7	100 0	
**Year of study** (%; undergraduate medical students only)		-	-	-	-
Year 1	13.0				
Year 2	26.3				
Year 3	18.3				
Year 4	30.9				
Year 5	10.7				
**MHAQ Score** (mean item score, SD)					
Total	2.8 (0.2)	2.7 (0.3)	2.8 (0.3)	2.8 (0.2)	2.7 (0.2)
Provision of high-value care	3.1 (0.3)	3.1 (0.5)	3.2 (0.5)	3.1 (0.4)	3.1 (0.4)
Integration of healthcare costs	2.7 (0.4)	2.6 (0.4)	2.7 (0.5)	3.0 (0.4)	2.7 (0.4)
Drawbacks of HVCCC	2.4 (0.4)	2.4 (0.4)	2.4 (0.5)	2.3 (0.5)	2.4 (0.4)

**Table 2:** Summary of cronbach’s alpha values for Maastricht HVCCC Attitude Questionnaire (MHAQ) subscales in medical students, interns, junior doctors, and senior doctors

	Medical student *N* = 262	Intern *N* = 80	Junior doctor *N* = 57	Senior doctor *N* = 16	Total *N* = 416
**MHAQ**					
Provision of High Value Care	0.71	0.63	0.72	0.68	0.69
Integration of Health Care Costs	0.72	0.77	0.76	0.79	0.74
Perceived Drawbacks of HVCCC	0.45	0.67	0.80	0.85	0.60

**Fig. 1:**
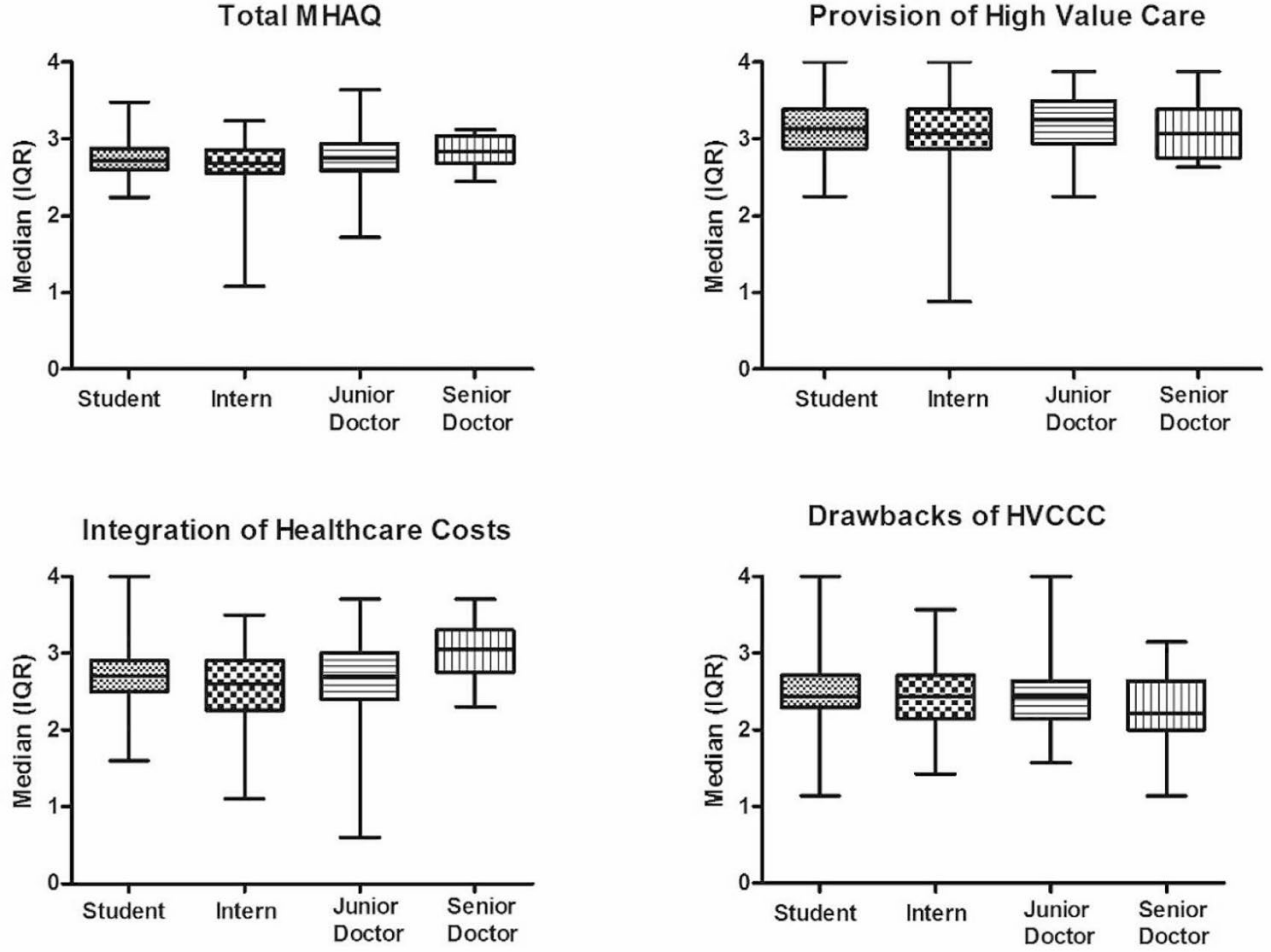
Median (IQR) item scores for MHAQ total and subscale scores in medical students, interns, junior doctors, and senior doctors

Significant group differences based on professional categories were observed for the “Integration of Healthcare Costs” sub-scale (H = 16.84, *P* = 0.001), where senior doctors exhibited significantly higher scores relative to interns or junior doctors (both *P* < 0.001), as well as students (*P* = 0.02) (See Fig. 1). No other difference in MHAQ score related to professional category was found. Within the medical student group, statistically higher scores for “Provision of High Value Care” (H = 13.62, *P* = 0.009) and “Integration of Healthcare Costs” (H = 9.87, *P* = 0.04) by an increase in programme year were found (Supplementary Data 2). Years since graduation among qualified doctors (i.e. excluding medical students) was weakly but significantly correlated with higher scores for MHAQ Total (rs = 0.19, *P* = 0.02) and the “Integration of Healthcare Costs” subscale (rs = 0.28, *P* = 0.001). Males demonstrated higher scores for MHAQ Total (U = 16,845, *P* = 0.001), “Provision of High Value Care” (U = 17,641, *P* = 0.007), and “Integration of Healthcare Costs” (U = 17,628, *P* = 0.007).

### Thematic analysis results

The consultants who participated in this study had between 1 and 24 years of experience in their field, averaging 15 years. The duration of the interviews varied, ranging from 14 min to 41 min, with an average length of 26 min.

**Fig. 2:**
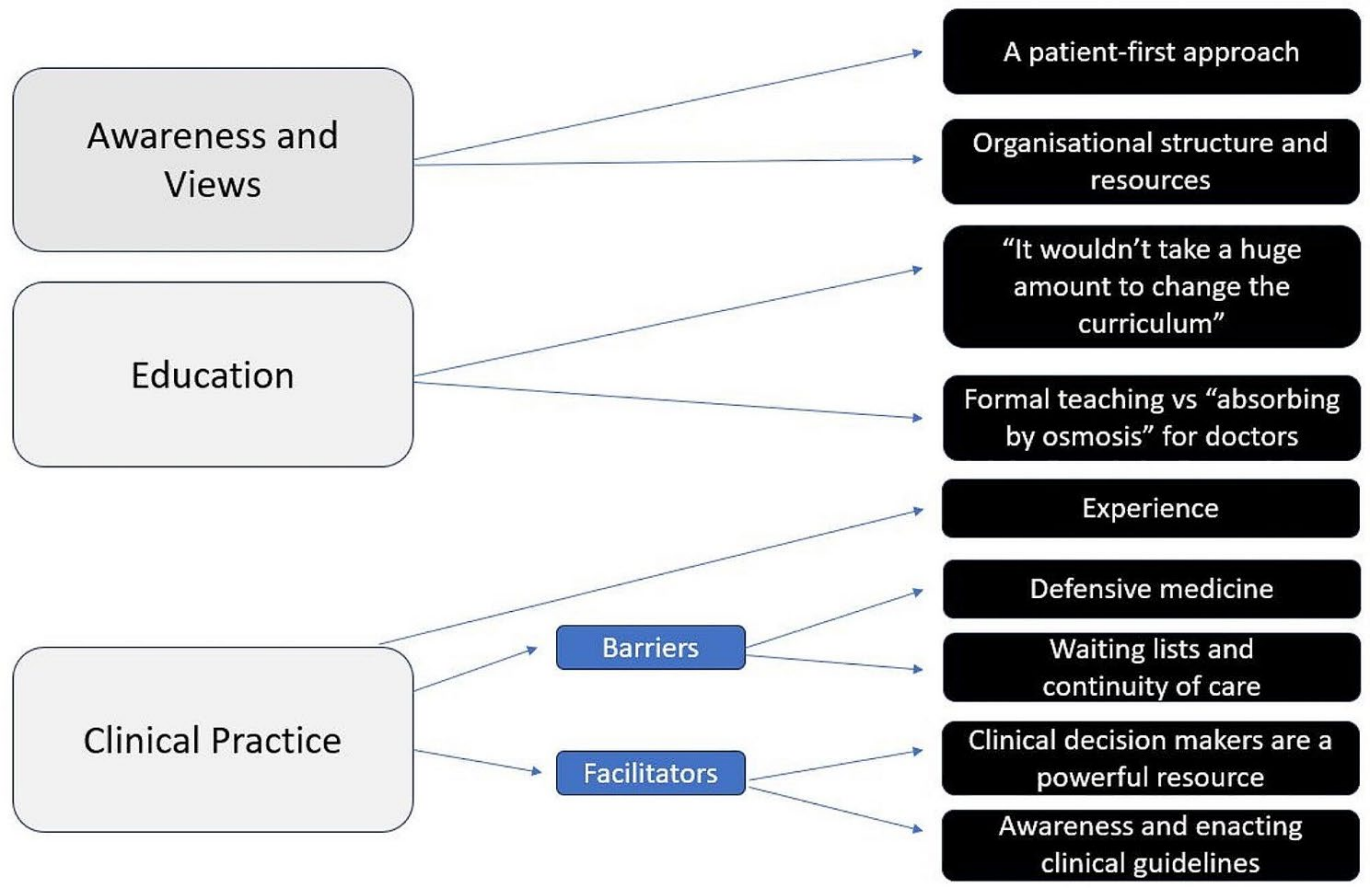
Superordinate and subordinate themes identified

Analysis of the data elicited three overarching themes. These include awareness and views of HVCCC, the role of education in the context of HVCCC, and the impact of HVCCC on clinical practice. A summary of the themes and subthemes constructed is available in Fig. 2.

### Awareness and views of HVCCC

Only two of those interviewed had previously heard of the term “high-value cost-conscious care,” however all were aware of the concepts that it entailed and agreed that it played a role in daily practice as a doctor. While the concept was universally understood, it was clear that HVCCC concepts held varying degrees of importance to the senior doctors involved, more specifically dealing with costs before the delivery of care.

### A patient-first approach

A common factor identified amongst interviewees was the point at which costs came to prominence in individual patient care. When providing care, costs only became a factor to consider when a specific avenue of care was unavailable due to cost or availability. Treatment unavailability caused a significant degree of frustration for doctors in situations where a specific avenue of management would provide value for the patient: “I don’t think we should have to sacrifice care based on costs, if we know that there is a cost-effective treatment for our patients in an ideal world, I feel we should be able to access that.” (Senior Doctor 6).

In some cases, specific interventions may be available under private healthcare but not from a public perspective, moving cost concerns from doctor to patient. Senior Doctor 2 felt that “you need to be mindful that certain patients won’t necessarily have care available to them publicly, and they won’t necessarily have the funds to procure it privately.” Waiting lists mean that by the time the care is available it may not be appropriate or relevant to a specific patient. Treating patients with a patient-first approach prevents “the problem of not delivering care.” Senior Doctor 2 further added, “the associated cost to the individual and the ultimate health care costs for that individual that will be borne by the state, if they don’t get initial treatment in an appropriate time.” In tandem with these views, several other participants mentioned initial value-based care via a patient-first approach as not delivering care may prevent unexpected associated costs such as re-presentation and admission.

### Organisational structures and resources

The overall structuring of clinical pathways and the utilisation of current healthcare expenditure was a recurring theme of interest among the interviewees despite no direct questioning on clinical governance. Efficiency and organisation were seen as key factors to improve value-based healthcare while reducing costs.

The public healthcare system in Ireland was seen as an “often resource-depleted environment.” (Senior Doctor 6). As a result, doctors sometimes felt they may “not be able to meet the goals for the patient within the constraint of the health service” (Senior Doctor 6).

A major difficulty with implementing changes that would impact clinical structures was the significant adjustments required to the healthcare system at an organisational rather than individual level: “The problem with that is, you’re talking about a kind of sea change and the shift in attitudes …. And a move away from ‘this is how we’ve always done it’ and perhaps looking at another way of doing things” (Senior Doctor 8).

Few solutions were offered to address these issues; however, innovation and implementation science were cited by doctors 1, 3, and 11 as mechanisms to provide the same or better care with less expenditure within the context of the current public healthcare system.

### The role of education in the context of HVCCC

Interviewees were asked how their own understanding of HVCCC was shaped, where they believe the current state of HVCCC education lies, and their ideas for undergraduate and postgraduate teaching.

Experience was again cited as a significant factor aiding doctors’ ability to provide HVCCC. Their education on costs in clinical practice generally stemmed from informal learning over time. They indicated that these concepts have become more of an issue throughout their training and learned informally while working: “I think as I have progressed from being a trainee to consultant and as now, I have a little bit more control over my own service, I think I am more conscious of the cost of treatments that we use and how certain treatments we use were very costly and replaced with other services that maybe gave us better value. That would be something I’d be interested in being able to influence if possible” (Senior Doctor 6).

### Integration of HVCCC into medical training

In general, doctors felt that HVCCC should feature principally during postgraduate training rather than at an undergraduate level. The principal reason cited was time constraints and the context of education in medical school. Senior Doctor 8 shared their opinions on the matter stating “I don’t think there’s any benefit at undergrad at all really, medical students have more important things to be thinking about than how much it costs to give something”.

Some interviewees believed it could form a small part of the curriculum in medical school without massively changing current undergraduate structures of teaching. They felt it could be integrated with the current teaching, introducing the concept of HVCCC and specific costs associated with clinical procedures, medications, or processes.

Several participants believed “it wouldn’t take a huge amount” to change the current curriculum in medical school, introducing concepts to build on in subsequent training. They discussed what undergraduate training could involve within the current curriculum, looking not only at the financial cost but the cost of illness to the patient and the value provided by good quality medical care: “I think the concept of cost-effectiveness and also measuring the effectiveness should be started at an undergraduate level, and then I think a deeper understanding of how you chose treatments and how you work out their cost effectiveness is probably a greater task for post-graduate training” (Senior Doctor 5).

Doctors interviewed felt that clinicians in postgraduate training schemes would derive the most benefit from formal HVCCC education as opposed to solely informal learning acquired through clinical practice.

This lack of a “fixed home” for HVCCC recurred throughout the interviews. Senior Doctors 6 and 8 shared some ideas on how to structure education of HVCCC values in postgraduate training.

“I think it could be part of a higher level, higher specialist training schemes, maybe some kind of module around healthcare economics or something along those lines might be beneficial at that point …. Maybe before people take on a consultant post, it would be a good idea to have an idea of cost and that kind of thing as opposed of only absorbing it by osmosis” (Senior Doctor 8).

“I think it should be a formal curriculum, maybe not-all focused-on cost consciousness but I think an overall curriculum on how and why we choose the treatments that we do and whether or not they’re effective or not and then how we measure the cost of them” (Senior Doctor 6).

These views agree with the idea of finding a “fixed home” for HVCCC education. Assessing the recurring themes that emerged, a common path to teach value and cost care emerged as seen by these senior doctors. This would include the integration of HVCCC into the current undergraduate curriculum, followed by formal teaching as a part of postgraduate medical training schemes to supplement knowledge, acquired through clinical practice.

### The impact of HVCCC on clinical practice

Examination of the interviews in relation to participants’ day-to-day clinical practice established numerous factors that facilitated or impeded HVCCC provision.

### Experience

Experience was a significant factor that could enhance and enable HVCCC over time, while lack of experience or clinical knowledge could be to the detriment of value for the patient or costs to the healthcare system.

Overall, doctors felt that experience was a major facilitator to HVCCC although they did recognise that it can be a “double-edged sword,” with a protocolised approach providing greater value and accuracy of care in slightly atypical presentations with one consultant reiterating that the ease of action associated with experience “may come at a cost for a minority” (Senior Doctor 3).

The inexperience of doctors, whether due to treating outside their specific specialty or being at the junior levels of postgraduate training was seen as an unavoidable factor that drove unnecessary testing: “The inexperience of the doctor probably leads into a patient safety approach …but the result is sub-optimal practice occurs, and the driver for that is either a very specific fear of criticism, or litigation” (Senior Doctor 3).

While experience in the overall workforce can be acquired by hiring experienced workers, this is not a readily available solution, highlighting the role of good clinical skill-based education that incorporates value and cost-conscious concepts.

### Barriers to HVCCC: Defensive medicine

The concept of “defensive medicine” was a recurrent topic brought up by participants when asked about factors that influence their daily clinical practice in a way that may not be cost-conscious, or value-based. Participants confirmed that fear of litigation underpins many investigations undertaken and may lead to unnecessary testing: “When investigations are being done there probably is a big medicolegal element to it….there can be a big variety between consultants and it’s down to people own fear of potential litigation if something is missed” (Senior Doctor 5).

### Barriers to HVCCC: Waiting lists and continuity of care

A common frustration noted amongst interviewees was identifying the ideal next course of action for a specific patient, but due to long waiting lists or lack of specific services, an avenue of management was not available or feasible to pursue. Situations like these may limit the value of care while increasing costs for the healthcare system and possibly the patient: “I think the main barrier in terms of cost is we can make a plan for our patients…but what they need to do that, or the supports that they need are often not possible from a cost point of view … often we are not able to meet the goals for the patient within the constraint of the public health service.” (Senior Doctor 6).

### Facilitators of HVCCC: Clinical decision makers as a powerful resource

Emphasis was placed on those staff with enough clinical experience to make decisions and progress care for the patient without excessive senior supervision: “Adding more frontline clinical staff at a senior decision-making level, because there’s no point in asking the senior house officer to go down there and sort out the ten patients in Accident and Emergency. That’s often what happens, but they’ll have to talk to the registrar who will have to talk to the consultants … it means you get rid of a lot of waiting in hospital …. Look at the patient’s pathway through the hospital and find ways to shorten waiting times for investigations and reviews” (Senior Doctor 8).

### Facilitators of HVCCC: Clinical guidelines

Clinical guidelines to aid decision-making in clinical practice were a recurring theme when interviewees were asked about the facilitators of HVCCC. Interviewees believed guidelines are particularly beneficial for doctors at a more junior level of training to reduce ambiguity in the progression of patient care.

Guidelines provide the background in which doctors can utilise their own clinical judgement to maximise the value of care provided to the patient while preventing unnecessary testing and procedures.

## Discussion

Medical students and qualified doctors showed moderately positive attitudes regarding HVCCC overall, as well as in each of the subscales. Seniority and experience (i.e., years since graduation) were both associated with more positive attitudes towards HVCCC, in particular as it related to the degree to which doctors should integrate costs in their daily clinical practice. Additionally, medical students demonstrated increasingly positive attitudes towards HVCCC as they progressed through the curriculum.

Generally positive attitudes regarding the provision of high-value care are congruent with findings reported in the literature [20]. Dyrbye et al. [27] found that 75% of doctors surveyed agreed that they have a responsibility toward cost-containment. Similarly, in their 2018 study comparing the attitudes of medical students and practicing doctors regarding cost-conscious care, Leep Hunderfund et al. [28] found that 86% of the respondents agreed that trying to contain healthcare costs is the responsibility of every doctor. They also reported that students and doctors both felt strongly that doctors should take a prominent role in limiting unnecessary testing. In the current study, particularly as it relates to differences in HVCCC between medical students in the preclinical cycle and those in the later clinical years, it is easy to see how the lack of experience and knowledge on the vast range of risks, benefits, and financial burden of investigations and management options, could lead to less clarity around their importance.

However, while 97% of American medical students surveyed felt that doctors should be aware of the costs of tests being ordered and of recommended treatments, only 76% of surveyed doctors indicated they were actually aware of these costs [28]. This level of awareness is quite high in comparison to the findings of other studies. Colla et al. [29] found that only 36.9% of participating doctors felt they had a good understanding of the costs of investigations and treatments in the healthcare system. So, while the data demonstrates that there are positive attitudes towards incorporating costs into practice, it does not tell us whether there is an understanding of the costs of care [30, 31].

A consensus was formed amongst senior doctors in this study concerning current and possible future HVCCC education strategies. In general, it was felt that incorporation of HVCCC values into the current undergraduate teaching, followed by a formal curriculum in postgraduate clinical training, as supplementation to informal knowledge gained through clinical practice would be valuable for doctors in training. There is extensive literature based on the US medical system where the concepts of HVCCC are a “seventh core critical competency,” meaning a formal curriculum is used in the certification and recertification of physicians [29]. Creating an equivalent “fixed home” for HVCCC training as part of each national or local competency framework for clinical effectiveness education could be beneficial in promoting the efficient use of healthcare budgets. However, this must be supported by an environment conducive to HVCCC concepts [21, 30, 31].

Questioning senior doctors on their everyday clinical practice revealed five main factors that may facilitate or obstruct the provision of HVCCC. These factors have been noted before in numerous qualitative and quantitative studies [11, 32–35]. Efficiency, continuity of care, and appropriately timed intervention were seen as facilitators of HVCCC in this study and may indicate a specific path to alleviate some concerns associated with clinical structures and governance as noted [36, 37]. However, it was unclear as to where efficiency targeting would be appropriate from the level of the individual doctor to the overall organisation. An exploratory study by Arnetz et al. [38] cites efficiency to achieve better patient satisfaction and reduce costs which are in line with the theme of a patient-first approach elicited in this study. Numerous studies cite a “triple aim”—improving the patient experience of care, improving the health of populations, and reducing the per capita cost of health care [7, 38]. A fourth aim— improving the healthcare provider experience has been proposed by some [39]. The efficiency of care in line with the above values may be a central way to explore current HVCCC delivery across national health systems in future studies.

In line with the MHAQ findings in both students and qualified doctors which highlighted experience as a factor mediating attitudes towards HVCCC, experience was cited in interviews as a necessary early barrier and late facilitator of HVCCC highlighting the role of both effective clinical and HVCCC education [13]. Senior doctors that can act as clinical decision-makers to promote both efficiency and quality of care are an encouraging product of this style of education and a noted HVCCC facilitator in this study. Recent studies from the Netherlands [30, 31], corroborated the importance of “expertise” to act with value and cost in mind.

Defensive medicine was the most common response when asked about barriers to HVCCC in this study. This is common amongst the literature pertaining to HVCCC [34, 35, 37]. Patel et al. describe 25 to 30% of healthcare expenditure as being due to the threat of liability and self-protection against potential lawsuits [21]. Clinical guidelines and awareness of these guidelines were cited as a component that could be utilised to reduce excessive costs due to defensive medicine. In keeping with this, doctors included in a qualitative study by Stammen et al. [30] deemed a clear institutional policy and associated well-defined clinical guidelines as an essential mediator of HVCCC.

This study has several limitations. One limitation of the questionnaire component is the large proportion of students or junior doctors in the sample population, leading to a sample skewed towards respondents with less clinical experience and familiarity with HVCCC principles in action. Additionally, the small number of senior doctors who completed the survey may impact the conclusions drawn from statistical comparisons with other participant groups. While interns were recruited from across the national intern training network, junior and senior doctors were all sampled from hospitals within Cork. Additionally, the sample may be biased as those who responded may have a particular interest in this area. As such, the data may not be representative of the attitudes or perceptions of doctors across Ireland. Shared findings between this study and current HVCCC literature suggest the reliability and accuracy of results. Additionally, the internal consistency reported here closely matches values previously reported by qualified doctors [19]. The sample also contained a relatively wide range of specialties to attain a suitable representation of the Irish healthcare system.

## Conclusions

There are overall positive attitudes regarding HVCCC, the provision of high-value care, and the integration of HVCCC principles into practice among this sample of medical students and hospital-based doctors. The generally positive attitudes indicate that interns and junior doctors in Ireland may be receptive to HVCCC training in the future. Experience plays an integral role in HVCCC, therefore early introduction to these concepts through formal and informal education may optimise practice within the current healthcare budget. Efficient care achieved through improving organisational structures, utilising clinical decision makers, reducing costs, and clear clinical guidelines should be promoted, allowing doctors to provide a “patient-first” approach.

### Electronic supplementary material

Below is the link to the electronic supplementary material.

Supplementary Material 1

## Data Availability

The authors declare that all the data and materials supporting the findings of this study are available within the article and are available from the corresponding author upon request.

## Abbreviations

HVCCC: High-Value Cost-Conscious Care
MHAQ: Maastricht HVCCC-Attitude Questionnaire
