# Supplementary material for: Implementing high-value, cost-conscious care: experiences of Irish doctors and the role of education in facilitating this approach
Source: BMC Med Educ. 2024 Jun 21;24:684. doi: 10.1186/s12909-024-05666-x (PMC11191173; doi:10.1186/s12909-024-05666-x)
Supplement: Supplementary file 1 — Supplementary Material 1 [file 12909_2024_5666_MOESM1_ESM.docx]

Supplementary Data 1: Semi-Structured Interview Template

**Consultant-level doctors’ perception regarding high-value cost-conscious care: a qualitative study**

- Prior to interview participants read information sheet and sign consent form
- Semi-structured interview format is explained

| **Theme** | **Main Questions** | **Essential Subtopics/Possible Subtopics**  **(If in bold essential subtopic)** |
| --- | --- | --- |
| Introduction | **Background Information**  “Would you like to tell me a little bit about your work | - When did you start working as a consultant?  - What specialty?  - What does your current role involve  - Previous roles |
| Theme 1 | **Awareness of HVCCC**  “Have you heard of, and what do you know about the term HVCCC”  **Give formal definition post identification of consultants understanding, identifying any changes to views**:  “High-value, cost-conscious care refers to care that aims to assess the benefits, harms, and costs of medical interventions, while aiming to add value for the patient in terms of their health, the doctor in terms of their resources used and the healthcare system as a whole”  **Indicate topics to be covered**:  “The main topics I would like to cover are the doctors role and your experience in providing value based healthcare while being aware of the associated healthcare costs, barriers or disadvantages to HVCCC and your views on education in relation to HVCCC.” |  |
| Theme 2 | **Providing Value-Based Care**  “What does providing value-based healthcare mean to you” | - Do you have any specific examples of working to provide value in your clinical practice.  **- Do you think it is the doctor’s responsibility to limit unnecessary tests or procedures?**   - If not, who’s responsibility is it to limit unnecessary tests or procedures?   - **Is it safe to limit unnecessary tests**   - Does the risk outweigh the benefit of ordering fewer tests?   - Any factors that cause you to order more tests?  - Are there any interventions you carry out that do not influence your management of a patient?  - **Does your experience influence the amount/types of tests you order?**   - If so, how has this changed over time? |
| Theme 3 | **Involvement of Healthcare Costs**  “How do healthcare costs influence your practice” | - Do you think a doctor should think about costs to the healthcare system in their practice   - Costs to society?   -**Is it unfair to make a doctor think of costs when managing a patient?**  - Do you think a doctor should consider costs for the patient?   - Is this easy/hard to bring up with patients? - Is it appropriate for a doctor to talk about the costs of care when discussing treatment options?   - **Are you aware of the costs of tests?**  **- Are you aware of the costs of prescribed medications?**  - Do physicians play a major role in driving healthcare costs or is there other factors.  - Should cost effectiveness data be used to determine treatments offered to patients?   - Are there any examples where a treatment is effective, but the costs may be prohibitive in you? |
| Theme 4 | **Issues with/Barriers to HVCCC**  “Is there anything that you feel acts a barrier to providing HVCCC”  “What would allow you to provide HVCCC more easily” | **- Does fear of litigation influence the tests/procedures you order?**  - Would discussing costs of care with the patient have a positive or negative influence on the doctor-patient relationship.  - Are there any resources that allow you to work with HVCCC in mind.  - Are intervention costs being visible when ordering tests/prescribing present   - If so, do you feel they influence your decisions   - Are there guidelines in place that allow you to work cost consciously |
| Theme 5 | **Educations role in HVCCC**  “Has HVCCC been taught to you, if so, how?” | - **What has your experience been with regards HVCCC education**   - Was it formal/informal teaching? - Any specific ways you were taught HVCCC - Did clinical role models act as a form of HVCCC education.   - Do you think you currently teach HVCCC, if so, how?   - Any methods of teaching you feel are effective/would be effective?   - **Where do you think HVCCC should be taught?**   - Should it be taught formally in college or in post graduate training? - What methods of teaching?   - Would reports on individual healthcare spending in accordance with guidelines be effective. |
|  | **Explore Opinions and Close**  “Is there anything else relating to HVCCC you want to talk more about”?  Debrief and Thanks. |  |

Supplementary Data 2: Median (IQR) total and mean (SD) item values for MHAQ total and subscale scores in medical students across the five years of medical school

|  | **Programme year** | | | | |
| --- | --- | --- | --- | --- | --- |
|  | **Year 1**  N=34 | **Year 2**  N=69 | **Year 3**  N=48 | **Year 4**  N=81 | **Year 5**  N=28 |
| **Median, IQR** | | | | | |
| Provision of High Value Care | 24 (3.3) | 24 (3.0) | 24 (4.0) | 26 (3.0) | 24.5 (2.8) |
| Integration of Health Care Costs | 26 (4.5) | 26 (5.0) | 27 (5.0) | 28 (4.5) | 28 (5.8) |
| Perceived Drawbacks of HVCCC | 18 (3.0) | 17 (3.0) | 17 (3.0) | 17 (3.0) | 16.5 (3.0) |
| Total | 66 (6.0) | 68 (7.0) | 68 (7.5) | 69 (6.5) | 67.5 (7.8) |
| **Mean, SD** |  |  |  |  |  |
| Provision of High Value Care | 3.0 (0.3) | 3.0 (0.3) | 3.1 (0.3) | 3.2 (0.4) | 3.1 (0.3) |
| Integration of Health Care Costs | 2.6 (0.4) | 2.6 (0.4) | 2.7 (0.4) | 2.7 (0.3) | 2.8 (0.4) |
| Perceived Drawbacks of HVCCC | 2.6 (0.4) | 2.5 (0.3) | 2.4 (0.4) | 2.4 (0.4) | 2.4 (0.3) |
| Total | 2.7 (0.2) | 2.7 (0.2) | 2.8 (0.2) | 2.8 (0.2) | 2.8 (0.2) |
